# Understanding anatomical plasticity of Argan wood features at local geographical scale in ecological and archaeobotanical perspectives

**DOI:** 10.1038/s41598-021-90286-4

**Published:** 2021-05-24

**Authors:** Jérôme Ros, Jean-Frédéric Terral, Marie-Pierre Ruas, Sarah Ivorra, Bertrand Limier, Mohammed Ater, Laure Paradis, Ahmed S. Ettahiri, Abdallah Fili, Jean-Pierre Van Staëvel

**Affiliations:** 1 Institut des Sciences de l’Evolution – Montpellier, CNRS, IRD, EPHE, Univ. Montpellier, Place Eugène Bataillon, 34095 Montpellier Cedex 5, France; 2 International Associated Laboratory / International Research Program (LIA/IRP, INEE-CNRS) EVOLEA, France-Morocco, Montpellier, France; 3 Archéozoologie, Archéobotanique: Sociétés, Pratiques Et Environnements (AASPE), CNRS, MNHN, CP56, 43 Rue Buffon, 75005 Paris, France; 4 INRAE, Centre Occitanie-Montpellier, 2 Place Pierre Viala, 34000 Montpellier, France; 5 Laboratoire Botanique Appliquée, Equipe Bio-Agrodiversité, Faculté Des Sciences, Université Abdelmalek Essaâdi, BP 2060, 93 030 Tétouan, Morocco; 6 Institut National Des Sciences de L’Archéologie Et du Patrimoine, Département d’archéologie islamique, Madinat Al-Irfane, Hay ar-Riyad, Angle Rues 5 et 7, Rabat-Instituts, 10100 Rabat, Morocco; 7 Faculté Des Lettres Et Des Sciences Humaines, Université Chouaib Doukkali - El Jadida, 2, Avenue Med Ben Larbi Alaoui, B.P. 299, 24000 El Jadida, Morocco; 8 UMR Orient Et Méditerranée, CNRS, EPHE, Collège de France, Université Paris, 1-Panthéon-Sorbonne, 27 Rue Paul Bert, 94200 Ivry-sur-Seine, France

**Keywords:** Forest ecology, Palaeoecology, Environmental impact, Biological techniques, Environmental social sciences

## Abstract

The emergence of the Argan tree as an agricultural, pastoral, cultural, economic and ecological keystone species in Southern Morocco is considered to be linked to the settlement of agropastoral communities that favored its expansion. Nevertheless, the use and exploitation of Argan tree is documented by both few medieval written sources and archaeobotanical studies, from a single location, Îgîlîz (Toughmart, Morocco), a famous medieval site of the Anti-Atlas Mountains. Therefore, data remain scarce regarding the type of Argan communities exploited at this period. In order to document this question, a quantitative eco-anatomical approach aiming to understand variations of wood characters involved in sap conduction and reserve storage, is developed from modern samples collected in the area of Îgîlîz. Results show that diameter of branches and environmental factors are the major parameters explaining plasticity of wood anatomical characters. Quantitative eco-anatomical features of Argan archaeological charcoal confronted to two statistical models, allow assessing both the diameter of the branches from which it derives and the agro-ecological conditions of tree growth and development. This preliminary study may be considered as a relevant and pioneering work for the understanding of the eco-history of the Argan tree, and of its use and exploitation during the past.

## Introduction

**Figure 1:**
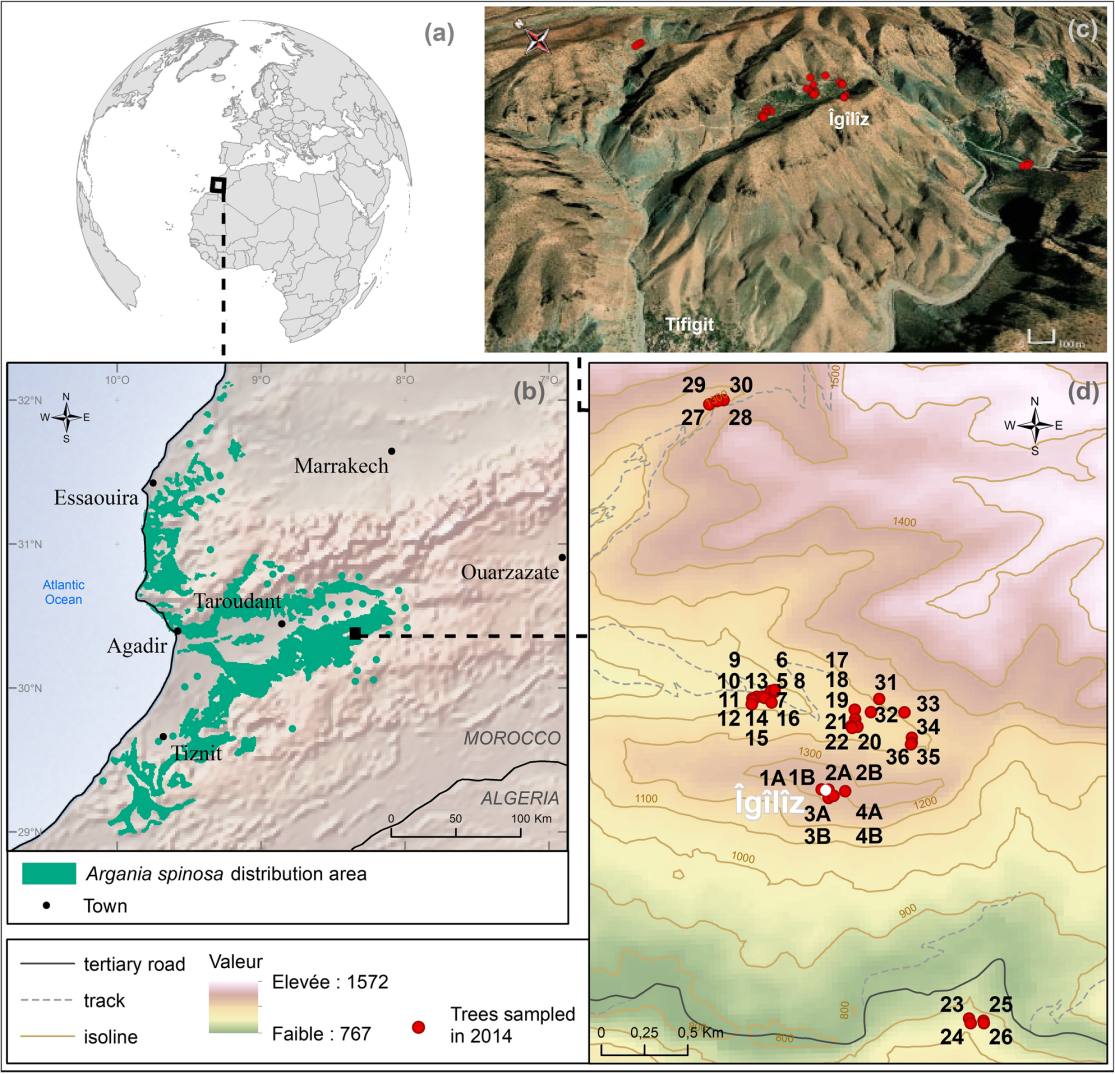
Location of the study area and sampled Argan trees. (**a**, **b**): Maps and Argan distribution area was drawn with the help of ressources in the public domain, ASTER GDEM (https://asterweb.jpl.nasa.gov/gdem.asp) and Natural Earth (https://www.naturalearthdata.com). (**c**, **d**): Geographical location of the sampled Argan trees is presented using © Esri softwares (ArcMap and ArcGlobe 10.7, Esri World Imagery). Maps were designed and drawn by Laure Paradis (ISEM 2020).

The Argan tree, *Argania spinosa* (L.) Skeels, Sapotaceae (‘Argân’ in Amazigh Berber language), is, along with the Atlas cedar (*Cedrus atlantica* (Manetti ex Endl.) Carrière, Pinaceae) and the two olive tree subspecies (*Olea europaea* L. subsp*. europaea* and *O. e.* subsp*. maroccana* (Greuter & Burdet) Vargas et al.*,* Oleaceae), the most iconic woody plants of Southwest Morocco. It is a xerophilous endemic species and the only representative of the tropical Sapotaceae family in Morocco. The Argan tree is distributed from sea level up to 1300–1500 m, from Safi (North) to the Drâa River (South) and isolated populations extend as far as Tindouf, well inside the Western Sahara (Fig. 1).

It forms particularly well-developed woodlands on the Essaouira and Souss plains, near the Atlantic coast, and rather open vegetation in the Anti-Atlas and the High Atlas area. The Argan woodlands may be considered as a transitional macro-ecotonal zone among two main bioclimatic and phytogeographic areas: (1) the thermomediterranean vegetations, further north, characterized by wild olives (*Olea europaea* ssp. *europaea* var. *sylvestris* (Mill) Lehr), the Mediterranean fan palm (*Chamaerops humilis* L., Arecaceae) and the Berber Thuya (*Tetraclinis articulata* (Vahl) Mast., Cupressaceae) communities; (2) Sahelo-Saharian shrublands reaching in the Argan area their septentrional boundary, dominated by Acacias and succulent species such as flat crown (*Vachellia gummifera* (Willd.) Kyal. & Boatwr., Fabaceae), resin spurge (*Euphorbia resinifera* O.Berg, Euphorbiaceae) and Swizzle Sticks (*Kleinia anteuphorbium* (L.) Haw., Asteraceae), respectively. The climate of the Argan tree zone benefits from temperate oceanic influences with annual precipitations between 150 and 400 mm and frequent fog throughout the year.

Its origin and biogeographical history remain enigmatic. The Argan tree is considered to have a Tertiary origin, period during which the coastal areas of the Southwestern Morocco were under tropical to subtropical influence. Its Pliocene range is thus supposed to be larger than the present distribution area^1,2^. However, this hypothesis is not supported by any tangible proofs. At the contrary, the palynological and palaeobotanical data of the late Pliocene and early Quaternary seem to show that the Argan tree occupied a more limited area than today. The Argan sporadic Quaternary pollen record pleads in favor of an ancient establishment, probably prior to the Quaternary, of its restricted endemism^3–6^.

A multiproxy analysis of marine sediment cores covering the last 2000 years showed that the Argan tree expanded very recently (around 150 BP) in Southeastern Morocco, while all woodland and shrubland communities continued their decline correlatively with the rapidly increasing population, pastoralism (mainly goat farming), deforestation and agriculture (olive cultivation)^7^. In his recent synthesis, Ballouche^8^ insisted on the importance of the goats that played a significant role in vegetation degradation while being an efficient agent of dispersion of the Argan tree by endozoochory. The dominance of the Argan woodland in Southwestern Morocco is thus mainly anthropogenic. Therefore, the Argan tree is the main element of a cultural landscape that may be considered as a socio-ecosystem. The tree, neither wild nor domesticated, is the mainstay of a production system for Argan oil and constitutes the major source of vegetable fat in the local traditional diet. Its wood is used as fuel and for construction. Its foliage and fruit pulp are an important source of feed for livestock^9–12^. In areas close to houses and in cultivated fields, Argan trees are protected and branches are cut only when hindering the passage of inhabitants, to develop the height of the tree, to increase fruit production, or to enable ploughing with mules or facilitate fallen fruit gathering^13^.

**Figure 2:**
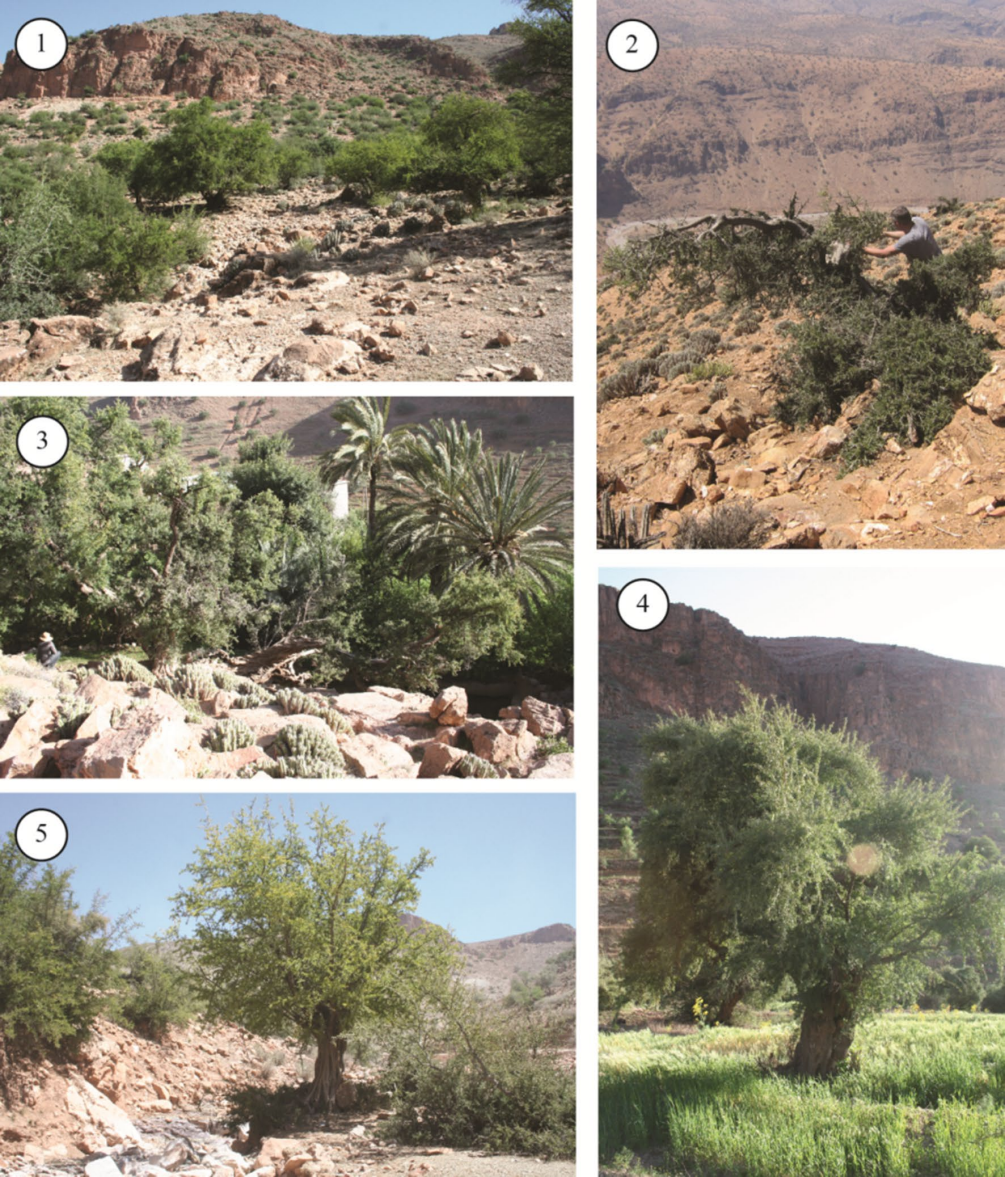
Different growing conditions and forms of the Argan tree in the study area: (1) Argan shrub-steppe; (2) Stunted small Argan tree frequently grazed by goats; (3) Argan tree in irrigated gardens; (4) Argan tree in a non-irrigated barley field; (5) Argan tree growing in a temporary stream bed (‘oued’) – All photographs were taken by J.-F. Terral and J. Ros.

The growth form of the Argan tree seems to be very variable according to local conditions. When managed and protected, for shade or fruit production, trees can become very large with well-developed crowns. On the contrary, when heavily browsed or pruned for fodder and fuel, they are reduced to stunted small trees and shrubs spreading on the rocky soil (Fig. 2).

Nonetheless, the history of Argan exploitation during historical times was, till recently, poorly documented but by very few medieval written sources. In the southern part of the country, excavations have been carried out at Îgîlîz for the past 15 years, as a part of a French-Moroccan cooperation research program directed by A. S. Ettahiri, A. Fili and J.-P. Van Staëvel. This program focuses on the origins of the tribal and religious movement that will found the Almohad empire (1147–1269), and aims to reconstruct the life of a peripheral Muslim mountain society in medieval and pre-modern times^14–16^. The archaeobotanical approach led on the site has revealed the first and oldest Argan remains in Morocco, resulting from the daily activities of medieval populations (exploitation of the Argan tree, fuel and fodder, and oil extraction). Numerous charred wood and seed shells of *Argania spinosa* predominated in a wide range of contexts, indicating the major role of this species in the tenth-thirteenth century economy. With the remains of the Argan tree, a diversity of crops (cereals, legumes and fruit trees) are attested. This rich and abundant carpological record have raised the question of techniques that were managed to maintain them in a landscape currently constrained by the semi-arid slopes^13,17^. For the present inhabitants of the neighboring village of Tifigit, an exceptional conservatory of the history and ethnobotanical observatory of pre-Saharan agrarian human communities located at 1200 m a.s.l. on the slope opposite the archaeological site, the Argan tree is an important resource as well, in domestic and subsistence activities. It is mixed with family staple crops growing in the irrigated and manured terrace soils. These practices allowed us to directly examine the management of the Argan grove and to observe its conditions of growth following different types of exploitation: irrigated manured trees in garden, non-irrigated manured trees exploited in association with crops, principally barley, unfertilized wild trees outside the village and the cultivated areas, all of them being grazed by livestock or not, depending on the will of the inhabitants (Fig. 2).

The first part of the study deals with a quantitative analytical approach on the influence of conditions of growth (irrigation, manuring, grazing) on the Argan wood anatomy, based on wood samples collected in the area of the Tifigit village and the Îgîlîz archaeological site (Table S1). In a second time, comparisons are made between the modern reference model and data derived from archaeological charcoal analyses (Table S2).

The objective of this work is threefold. First, it is intended to provide a detailed guideline of all stages of the methodological and analytical procedure of quantitative eco-anatomical analysis including preliminary tests such as the determination of the number of measurements required for an optimal assessment of each anatomical character and the reproducibility of measurements. Secondly, taking into consideration the age of the wood as an important factor of anatomical variability, this study attempts to assess, at the local geographical scale, the anatomical plasticity of Argan wood, in order to distinguish on the basis of quantitative anatomical features different Argan trees growing under distinct ecological conditions. Finally, the study aims to reconstruct the growth conditions and management of the Argan tree during medieval times at Îgîlîz, by confronting quantitative anatomical data from analysis of archaeological charcoal fragments to the eco-anatomical statistical model previously obtained.

## Results

Preliminary approaches and tests (determination of the number of required measurements for an optimal assessment of anatomical characters and measurement reproducibility) to quantitative eco-anatomical analyses carried out on current and archaeological Argan charcoal are presented in supplementary information (Fig. S1).

### Variation of wood anatomical features according to the diameter of samples

Quantitative data acquired and used for multivariate statistical analysis (PCA) are summarized in Table S3. The first principal components (i.e. coordinates of modern wood samples in the first axis) of the PCA are extracted from the results of the PCA. As the first principal components (PC1) of the PCA express the greatest variance while the diameter (DIA) is supposed to be the major parameter of wood character variability, the correlation among PC1 and DIA is tested.

**Figure 3:**
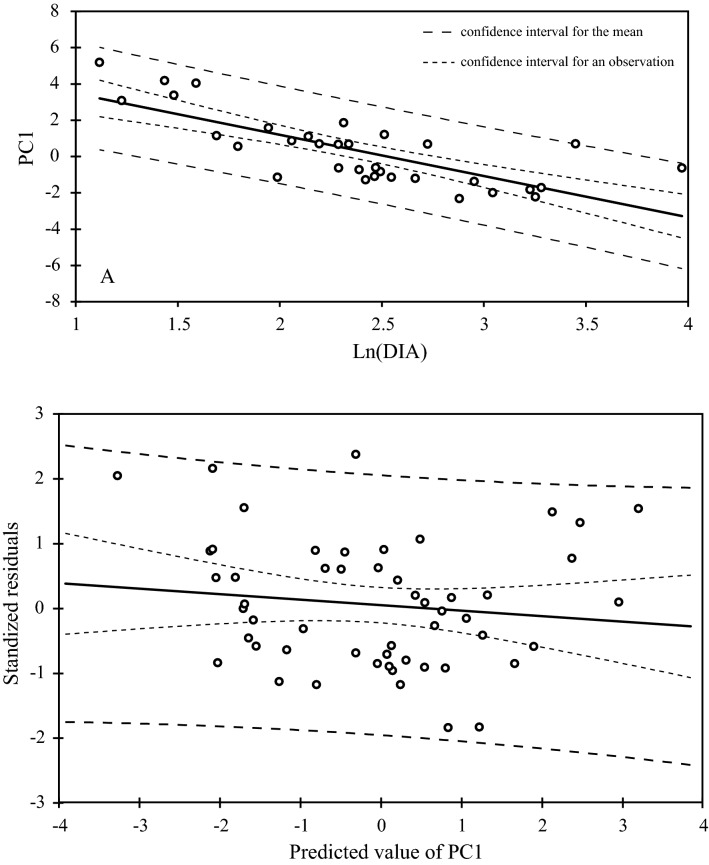
Linear regression model explaining the variation of PC1 according to the diameter of branch (caliber) (PC1 = f(Ln(DIA)) and diagram of the standardized residuals of the model.

Diameter values were previously Log-transformed (Napierian logarithm) to satisfy the requirement of normality and homoscedasticity (Shapiro–Wilk normality test: W = 0.98, *P* value = 0.51). Correlation between PC1 and sample diameter appears to be significant (Kendall correlation test: r = −0.761, *P* value < 0.0001). Then, variation of PC1 in relation to wood sample caliber is modelled with a least squares linear regression model (Fig. 3): PC1 = 5.73–2.27Ln(DIA). After examining the plot of residuals, this model satisfies the assumptions made prior to its establishment: non-correlation between residuals and adjusted values, sum of residuals equal to 0 and normality of residuals (w = 0.97, *p* < 0.24).

**Figure 4:**
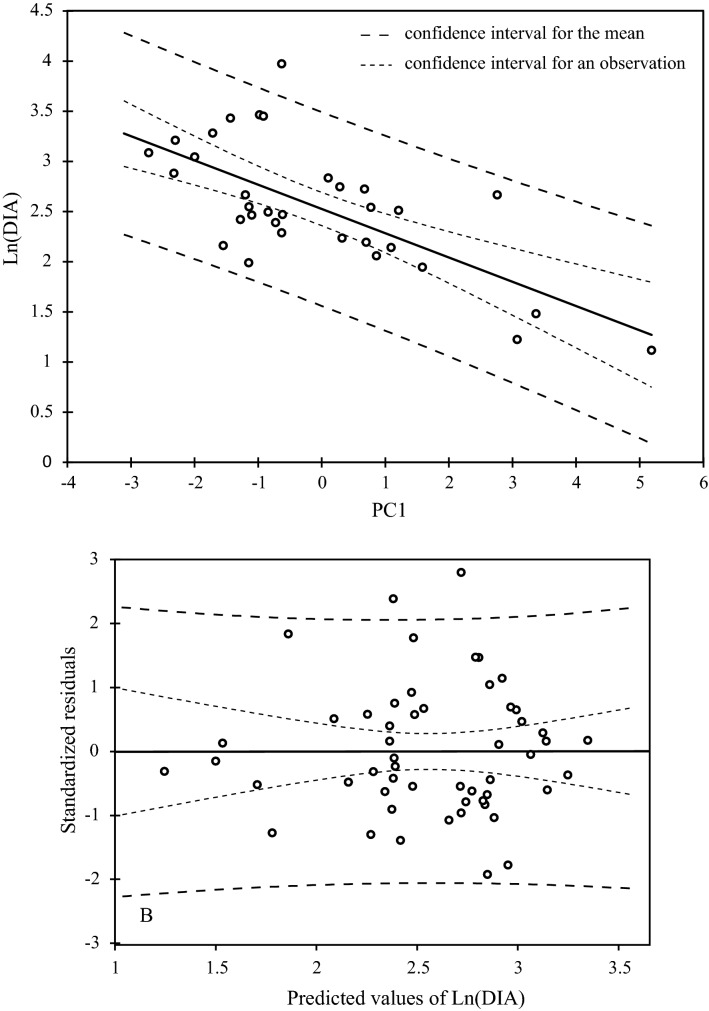
Linear predictive model of the branch diameter in relation to PC1 (Ln(DIA) = f(CP1)) and diagram of the standardized residuals of the model.

Since we know that PC1 varies in relation to wood sample diameter, we have developed a predictive linear regression model aiming to estimate the diameter of a branch (Fig. 4) (r = -0.69, *P* value < 0.0001). Then, the coordinate in the PC1 of the archaeological specimens have been integrated in the model to infer the diameter of a branch they came from. However, as it is impossible to know the position of the charred sample from which it originates, the diameter predicted by the model corresponds to a minimum diameter. This least squared linear regression model satisfies the suppositions made prior to its establishment: homoscedasticity, non-correlation between residuals and adjusted values, sum of residuals equal to 0 and normality of residuals (Shapiro–Wilk normality test: W = 0.97, *P* value = 0.24) (Fig. 4): Ln(DIA) = 2.53–0.24 PC1.

### Variation of wood anatomical features according to environmental parameters

**Figure 5:**
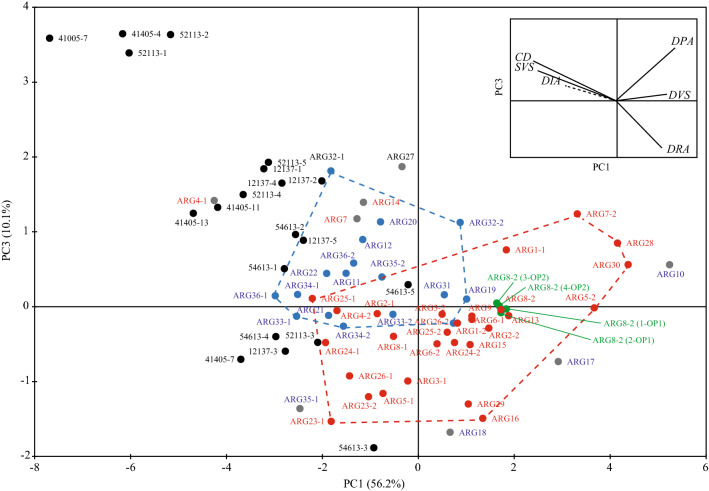
PCA analysis biplot 1–3 (66.3% of variance) showing a trend from samples of Argan trees growing in wild conditions (in red) to samples from trees in cultivated conditions (in blue). The grey dots represent the samples that do not match to the expected pattern of distinction. Test samples used to test measurement errors and repeatability are shown in green. Archaeological charcoal fragments used as additional statistical individuals are represented in black.

No influence of environmental parameters on wood anatomy has been demonstrated from the examination of distribution of individuals in the plane 1–2 of the PCA. As noticed above, the first axis and, at a lesser extent, the second axis, show variations related to the diameter of samples. But a trend from samples collected on trees growing in uncultivated (wild) conditions to samples from trees located within the local agroecosystem of the village of Tifigit seems to draw on the plane 1–3 (Fig. 5).

This trend appears to be explained by the axis 3 (10.1% of the variance) which seems to express variations in density of axial parenchyma (DPA) and wood ray density (DRA). Samples from trees growing in uncultivated (wild) zones are characterized by lower values of DPA and a higher DRA than samples collected on trees growing in cultivated areas. Nevertheless, 8 individuals do not match to the pattern shown by the PCA. The possible causes of their surprising positioning will be discussed later.

### Modeling of the minimum diameter of branches from which archaeological charred samples were produced

Coordinates in the first PCA axis (CP1) of the archaeological samples considered as additional statistical samples were applied to the predictive regression model (Fig. 4). Considering the confidence interval (at 95%), reconstruction of the minimum branch diameter was obtained by direct estimation from the predictive model (Table 1). The estimates appear relatively accurate when they are obtained by interpolation. But, it is not the case with higher values obtained by extrapolation. However, the results show that the majority of reconstituted diameters are below 50 mm and may be attributed to secondary small branches and twigs used for firewood (Gate 1-entry 1, Latrine and Fireplace). The highest modeled diameter values are those of the plank. They seem to correspond to the function and the size of the archaeological object that has been cut in one piece from the trunk of a tree. It can also be noted that the concentrated charcoal assemblages (Gate 1—entry 1 and Fireplace) gave equivalent diameter values. These charcoal fragments could come from the same branch, rather small in diameter.

**Table 1:** Predicted value of branch diameter (mm) assessed using linear regression analysis.

Stratigraphic unit	Archaeological context or object	Charcoal accession number	Predicted value of branch diameter (mm)	
			Mean	Confidence interval (at 95%)
12137	Gate 1-Entry 1	12137-1	27.20	[20.12–36.80]
		12137-2	20.32	[16.83–24.54]
		12137-3	24.43	[18.84–31.68]
		12137-4	24.84	[19.03–32.43]
		12137-5	22.30	[17.81–27.92]
41005	Plank	41005	80.05	[38.95–164.49]
52113	Latrine	52113-1	53.66	[30.49–94.43]
		52113-2	43.52	[26.83–70.62]
		52113-3	20.75	[17.04–25.25]
		52113-4	30.21	[21.45–42.54]
		52113-5	26.59	[19.84–35.64]
41405	Beam	41405-4	55.40	[31.10–98.71]
		41405-7	30.56	[21.60–43.23]
		41405-11	34.33	[23.20–50.81]
		41405-13	38.82	[25.01–60.26]
54613	Fireplace	54613-1	24.53	[18.89–31.87]
		54613-2	23.20	[18.25–29.49]
		54613-3	15.64	[14.34–17.06]
		54613-4	25.63	[19.40–33.86]
		54613-5	13.16	[12.90–13.43]

### Assessment of the ecological situation of exploited medieval Argan trees

Measurements of anatomical characters from archaeological charcoal were confronted as additional individuals to the reference model provided by the PCA. Distribution of archaeological samples in the principal plane 1–3 showed analogies with modern Argan samples (Fig. 5). The following samples (41408-7, 56613-4, 12137-3 and 52113-3) seem to possess the features of Argan growing in wild conditions. The sample number 54613-5 located in the overlapped of the two scatter plots could not be affiliated to a precise group. The other archaeological samples, characterized by a high value of axial parenchyma density (DPA) and a low wood ray density (DRA), originate from Argan trees growing in agricultural conditions.

## Discussion and conclusion

Previous studies have shown the major importance of age (or diameter of stem, branch or trunk) on the size of wood characters^18–21^. Through the negative correlation between branch diameter and coordinates (PC1) of samples from the PCA carried out on the basis of eco-anatomical data explaining mostly by ‘Vessel density’ (DVS) and ‘Density of wood fenestrated zones’ (DWF), our study pinpoints the relationship between anatomy and the branch age (Fig. 3). Young branches and twigs are thus characterized by a high density of vessels and fenestrated zones that, in an eco-functional perspective, may be interpreted as a higher level of sap conduction security^22^. In that case, the cambium functioning favors the establishment of wood with numerous and rather narrow vessels (low-porosity). In the case of a breakdown of sap conduction (cavitation), the contiguous vessels will be able to take over and allow the sap to reach the leaves at the apex of the last growth units. Moreover, the fenestrated zones are small and correlatively, the cells implied in the reserve storage (horizontal and axial parenchyma) are numerous. Antagonistically, the fibers filling the fenestrated zones are fewer, thus diminishing the support function of the stem and young branch wood. In the case of branches with increasing diameter and perimeter, characterized by a conduction limited to the last growth rings, the decrease in density of vessels and fenestrated zones linked to an increase of vessel surface area, shows that the sap conduction function of wood relies more on the efficiency than on the safety. The increase of sap efficiency allows conducting the sap, more efficiently to the upper sections of the tree.

In semi-arid and arid regions, as the Îgîlîz and Tifigit area, water resources in pastoral land are scarce and difficult to be detected. A careful examination of the landscape before sampling the trees allowed us to detect the possible presence of water: in the areas closest to villages and associated to irrigated gardens, most often built around or on travertine edifices (calcareous formations edified by precipitation of carbonates from hard water, mainly spring water or highly mineral rivers) and the beds or banks of generally intermittent streams that grow during severe thunderstorm episodes. However, the existence of a dry watercourse does not guarantee the presence of water in the soil, since it is rapidly drained and evaporated. Unfortunately, a small part of the rainfalls is thus absorbed by the soil. In the areas further away from villages, the water resources available to plants are very uncertain. Although adapted to conditions of intense xericity, the Argan tree can have an extremely developed root system that allows it to draw water from the deepest layers of the soil, as we have seen on some individuals where a large part of the root system was uncovered by a landslide.

In our study, a variation pattern of two anatomical characters ('Density of axial parenchyma' (DPA) and 'Ray density' (DRA)) contributes to distinguish samples issued from Argan trees growing under wild conditions from those from cultivated areas. The latter are characterized by a highly developed parenchyma vertical system (parallel to the conduction system) of cells, whose exclusive function is the reserve storage, while the horizontal system (rays) ensuring not only reserve storage but also the horizontal conduction of sap among vessel groups, seems to be weaker. Therefore, it results that growing conditions (elimination of competitors, addition of organic fertilizer and irrigation) unbalance the ratio reserve storage / use of nutrient in favor of the storage. Conversely, under more stressful, wilder conditions, Argan trees rather optimize horizontal conduction to the detriment of nutrient storage, which may be attributed to a strategy of sap conduction safety. Seven samples out of 53 analyzed samples (13.2%) seem not to match with this variation model. Trees growing in the bed or banks of intermittent streams (ARG14 and ARG27 samples) seem to benefit from a substantial water supply. For this reason, anatomical features of these samples, convergent to cultivated Argan trees such as the ARG7 and ARG10 samples, are driven by unsuspected water resources. On the other hand, trees from non-irrigated cultivated areas (barley field) on which the ARG17, ARG18 and ARG35 samples were collected, show anatomical features of wild individuals. In these cases, trees deeply rooted do not seem to take advantage of cultural practices and of the organic fertilizers (animal dung) spread on the soil of cultivated terraces. Finally, the possible outlying anatomical features of the ARG7 sample may probably be due to measurement errors, undetected during the implementation of preliminary tests.

From a bioarchaeological point of view, the minimal diameter of the branches, reconstructed on the basis of the linear prediction model applied to quantitative eco-anatomical features of archaeological samples varies from 1 to 9 cm, with a majority ranging between 1.2 and 5 cm (75% of the individuals). Although this result is to be considered with caution, since the branches may have been of higher diameter, it seems that the Argan wood used as fuel rather comes from the cutting of branches for firewood, the gathering of dead wood, or from the gathering of branches whose leaves have been previously cut for fodder.

75% of archaeological samples (N = 15) may be affiliated to Argan trees under cultivated conditions, whereas 4 samples seem deriving from individuals growing in wild conditions. Thus, it appears that Îgîlîz inhabitants have mostly used wood issued from Argan trees growing and exploited in the agro-horticultural environment located in the Îgîlîz mountain itself, and/or a few hundred meters below the settlement. It is very likely that the Tifigit garden and terraces set up around a spring allowing to irrigate the village, already existed during the Middle Ages. Nevertheless, in the current stage of research, we have no tangible archaeological evidence of the existence of a settlement at this location. While it was shown that the Argan tree was the basis of the daily economy and agro-sylvo-pastoral system, our results thus bring new data about the past exploitation of this emblematic fruit tree.

Tested with success on the olive^23,24^ and the grapevine^20,25^, the quantitative eco-anatomical method brings for the first time results on anatomical plasticity of Argan wood, on the ecological and functional signature of anatomical features and on the medieval exploitation of Argan wood as fuel. The preliminary application of this method to 20 charred wood fragments from Îgîlîz (eleventh-thirteenth century AD—Toughmart, Anti-Atlas, Morocco) allows to infer the diameter of branches used as firewood, mostly less than or equal to 5 cm. It also shows that the exploited Argan trees derive for the most part from cultivated areas (garden or cultivated terraces).

Finally, this study opens new and fascinating ecological, palaeoecological, bioarchaeological, historical, biogeographic and palaeoclimatic perspectives, that will be examined on the basis of a larger reference collection including samples from different diameters and various ecological contexts, from the ocean coast to the continental and mountainous areas.

## Methods

### Sampling, preparation and treatment of modern reference material

A total of 53 modern wood samples were analyzed. The modern reference samples were collected in 2014 during the annual archaeological field mission, from 36 individuals (Table S1). For some trees, two wood samples of different diameters were collected in order to take into account anatomical variability within individual.

The collected individuals showed different conditions of growth described in the introduction section and detailed in the Table 1. With the agreement of the Tifigit inhabitants and local authorities, wood sampling was achieved but samples were not collected from trunks, to avoid injuring trees of major symbolic, ecological and economic importance. Only section samples with perfect axial symmetry were retained to avoid any impact of biomechanical constraints (formation of reaction wood) on wood characters.

Once collected, the samples were air-dried during a month at the laboratory. The samples were separately wrapped in tin foil and buried in the sand and then charred without oxygen, at 450 °C for 15 to 20 min depending on the size of the sample. As a result, samples were enriched in carbon (content > 90%)^20,26^, reached their maximal shrinkage^27^, and thus are considered to become morphologically comparable to charcoal produced in medieval fires^27–31^. The minimum and the maximum diameter of wood samples were measured (mm) using a digital measuring calliper before and after carbonization. The diameter used in the following analyses is the mean of the two measurements carried out before carbonization.

### Archaeological material

Twenty archaeological charcoal fragments of Argan tree identified during a previous analysis session^13^ were included in this study (Table S2). All the Argan charcoal fragments were collected in the medieval archaeological deposits of Îgîlîz^13^. They come from various contexts, for the most part from living units, and belong to the period of highest human activity at the site, between the late 11th and early thirteenth centuries.

### Quantitative eco-anatomical analysis of wood applied to the Argan tree

The approach consists in measuring constitutive elements of wood and aims to understand variations according to intrinsic (inferred by the branch diameter mainly age of tree^18^, linked to the existence of growth rings that are often difficult to distinguish) and environmental parameters affecting the cambial activity and thus, rate of growth and wood development^20,28–30^. This high resolution analysis of wood structure, particularly of conductive elements, allows addressing numerous questions that have been successfully solved in the case of the olive tree and the grapevine, such as phenology, ecology, climate, impact of human activities and agricultural practices^20,24,25,31–33^.

**Figure 6:**
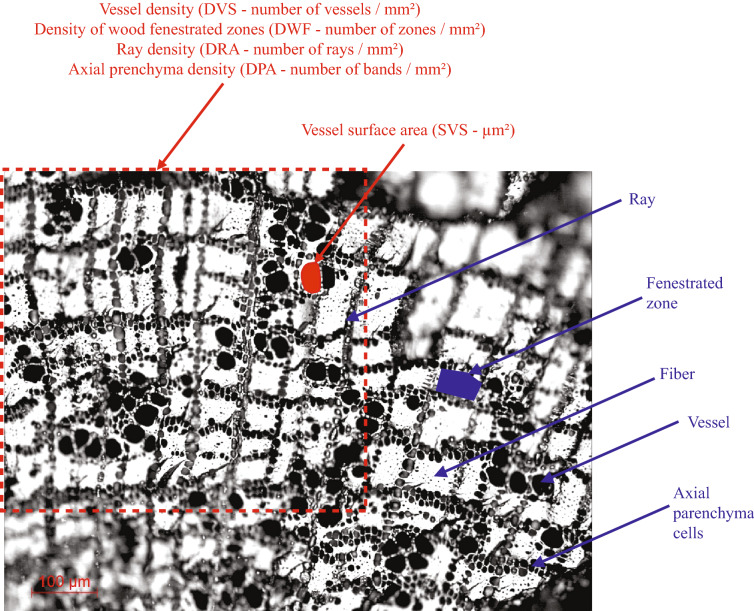
Wood anatomical features of the Argan tree (in blue) and measured anatomical characters (in red).

*Argania spinosa* wood is diffuse-porous with a dendritic and diagonal arrangement of vessel elements in transversal section^34^. The axial parenchyma bands are in tangential alignment and composed of multicellular strands. In radial alignment, woody rays are 1–3 cells wide and of heterocellular composition (Fig. 6).

To apply a quantitative eco-anatomy approach to the Argan tree, both modern charred samples and archaeological charcoal are broken manually in transverse anatomical section. The following wood constitutive elements and anatomical characters related to sap conduction and reserve storage are observed and measured under a reflected-light microscope connected with an image analysis system (DFC300 FX Leica camera and LAS Leica software) (Fig. 6): (1) vessel density (DVS—number of vessels / mm^2^), (2) vessel surface area (SVS, µm2), (3) ray density (DRA—number of rays / mm^2^), (4) axial parenchyma density (DPA, number of bands / mm^2^), (5) Density of wood fenestrated zones bordered on one side by the radial alignment of axial parenchyma cells and on the other side, tangentially, by rays (DWF—number of fenestrated zones / mm^2^).

These anatomical features were measured several times (see ‘Statistical analyses’ section) following radial lines from the cambium inwards the sample and crossing a small number of growth rings (i.e. functional rings from a sap conduction point of view). Moreover, the hydraulic conductivity or vascular conductivity (CD) was assessed using the following formula: CD = (SVS/π)^2^/DVS (after^32,35–37^). Finally, the ratio ‘Conductive surface / total wood area’ (SC) was calculated.

### Statistical analyses

In order to determine the number of measurements required for an optimal assessment of anatomical features, a rarefaction method was carried out from the analysis of test wood samples. For each one, repeated measurements of anatomical characters (Surface vessel area (SVS), Density of vessels (DVS), Ray density (DRA), Axial parenchyma density (DPA) and Density of wood fenestrated zones (DWF)) were performed following the aforementioned method and the cumulative mean value was then calculated for each character^20,29^. For each test sample and anatomical character, the number of measurements of a character required for an optimal assessment was quantified as the minimum number of measurements required to stabilize the mean value (rarefaction curve or cumulative mean curve).

Furthermore, different measurement sessions were carried out with the aim of testing possible errors and reproducibility of measurements taken by one or various observers, respectively. The data sets produced were tested using the PCA performed to evaluate the Argan anatomical variability. The ARG8-2 sample was used as test sample. In addition to the initial measurements. The ARG8-2 sample was analyzed 4 times: twice by one operator (ARG8-2 (1-OP1) and ARG8-2 (2-OP1)) and twice by another (ARG8-2 (3-OP2) and ARG8-2 (3-OP2)) at different times. The additional data were incorporated into the PCA as additional individuals for comparison with initial anatomical features of ARG8-2.

After showing that measurement errors have no impact on the validity of results and the measurements are reproducible, quantitative eco-anatomical data were processed using a principal component analysis (PCA) in order to evaluate anatomical plasticity in the reference modern material, to appreciate relationships between characters and wood sample caliber and to confront archaeological data to the reference model. PCA was applied on 53 reference modern samples and 7 quantitative variables (anatomical characters) to: (1) validate the hypothesis that there is a significant relationship between the size of the branch and anatomy, as previously demonstrated by analyses of wood development and structure^18,20,38^ and dendrochronology^39^; (2) identify the anatomical characters most affected by the age of the branch and, in that case, model the ‘anatomical characters—caliber of the branch’ relationship; (3) develop predictive model that might estimate the minimum branch caliber from eco-anatomical data of archaeological charcoal.

Finally, data from analysis of the 20 archaeological charcoal fragments were included in PCA as additional statistical samples. They do not contribute to the development of the reference model, but are compared to the modern reference samples in order to infer the ecological conditions under which Argan trees grew during the Middle Ages.

## Supplementary Information

Supplementary Information.
