# Supplementary material for: Understanding anatomical plasticity of Argan wood features at local geographical scale in ecological and archaeobotanical perspectives
Source: Sci Rep. 2021 May 24;11:10830. doi: 10.1038/s41598-021-90286-4 (PMC8144426; doi:10.1038/s41598-021-90286-4)
Supplement: Supplementary file 1 — Supplementary Information. [file 41598_2021_90286_MOESM1_ESM.docx]

**Understanding anatomical plasticity of Argan wood features at local geographical scale in ecological and archaeobotanical perspectives**

Jérôme Ros^1,2^, Jean-Frédéric Terral^1,2^, Marie-Pierre Ruas^3^, Sarah Ivorra^1,2^, Bertrand Limier^1,2,4^, Mohammed Ater^2,5^, Laure Paradis^1,2^, Ahmed S. Ettahiri^6^, Abdallah Fili^7^, Jean-Pierre Van Staëvel^8^

1. Institut des Sciences de l’Evolution – Montpellier, Univ. Montpellier, CNRS, IRD, EPHE. Place Eugène Bataillon, 34095 Montpellier Cedex 5, France.

2. International Associated Laboratory / International Research Program (LIA, INEE-CNRS) EVOLEA, France-Morocco.

3. Archéozoologie, Archéobotanique : Sociétés, Pratiques et Environnements (AASPE), CNRS, MNHN, CP56, 43 rue Buffon, 75005 Paris, France.

4. INRAE, Centre Occitanie-Montpellier. 2 Place Pierre Viala, 34000 Montpellier (France).

5. Laboratoire Botanique Appliquée, Equipe bio-Agrodiversité, Faculté des Sciences, Université Abdelmalek Essaâdi. BP 2060, Tétouan 93 030, Morocco.

6. Institut National des Sciences de l'Archéologie et du Patrimoine, Département d'archéologie islamique, Madinat Al-Irfane, Hay ar-Riyad, Angle rues 5 et 7, Rabat-Instituts, 10100 Rabat, Morocco.

7. Faculté des lettres et des sciences humaines, Université Chouaib Doukkali - El Jadida. 2, avenue Med Ben Larbi Alaoui, B.P. 299, 24000 El Jadida, Morocco.

8. UMR Orient et Méditerranée, Université Paris 1-Panthéon-Sorbonne, CNRS, EPHE, Collège de France. 27 Rue Paul Bert, 94200 Ivry-sur-Seine, France.

**Supplementary Table S1.** Presentation of modern reference samples.

| Argan tree | Latitude | Longitude | Altitude (m)^1^ | Growing conditions^2^ | Number of samples |
| --- | --- | --- | --- | --- | --- |
| ARG 1 | 30.39943 | -08.36553 | 1354 | 2 | 2 |
| ARG 2 | 30.39944 | -08.36590 | 1354 | 2 | 2 |
| ARG 3 | 30.39911 | -08.36528 | 1354 | 2 | 2 |
| ARG 4 | 30.39935 | -08.36467 | 1354 | 2 | 2 |
| ARG 5 | 30.40446 | -08.36861 | 1080 | 1 | 2 |
| ARG 6 | 30.40458 | -08.36853 | 1080 | 1 | 2 |
| ARG 7 | 30.40453 | -08.36836 | 1080 | 1 | 2 |
| ARG 8^3^ | 30.40463 | -08.36834 | 1080 | 1 | 2 |
| ARG 9 | 30.40427 | -08.36927 | 1067 | 4 | 1 |
| ARG 10 | 30.40418 | -08.36951 | 1067 | 4 | 1 |
| ARG 11 | 30.40410 | -08.36946 | 1067 | 4 | 1 |
| ARG 12 | 30.40389 | -08.36954 | 1067 | 4 | 1 |
| ARG 13 | 30.40427 | -08.36901 | 1073 | 5 | 1 |
| ARG 14 | 30.40423 | -08.36887 | 1073 | 5 | 1 |
| ARG 15 | 30.40422 | -08.36887 | 1073 | 5 | 1 |
| ARG 16 | 30.40398 | -08.36850 | 1073 | 5 | 1 |
| ARG 17 | 30.40360 | -08.36417 | 1088 | 3 | 1 |
| ARG 18 | 30.40311 | -08.36415 | 1088 | 3 | 1 |
| ARG 19 | 30.40289 | -08.36419 | 1088 | 3 | 1 |
| ARG 20 | 30.40271 | -08.36403 | 1088 | 3 | 1 |
| ARG 21 | 30.40274 | -08.36436 | 1088 | 3 | 1 |
| ARG 22 | 30.40265 | -08.36431 | 1088 | 3 | 1 |
| ARG 23 | 30.38750 | -08.35821 | 939 | 1 | 1 |
| ARG 24 | 30.38726 | -08.35813 | 939 | 1 | 2 |
| ARG 25 | 30.38738 | -08.35746 | 939 | 1 | 2 |
| ARG 26 | 30.38725 | -08.35744 | 939 | 1 | 2 |
| ARG 27 | 30.41951 | -08.37176 | 1276 | 5 | 1 |
| ARG 28 | 30.41972 | -08.37100 | 1276 | 5 | 1 |
| ARG 29 | 30.41968 | -08.37134 | 1276 | 5 | 1 |
| ARG 30 | 30.41969 | -08.37130 | 1276 | 5 | 1 |
| ARG 31 | 30.40416 | -08.36290 | 1197 | 3 | 1 |
| ARG 32 | 30.40347 | -08.36333 | 1197 | 3 | 2 |
| ARG 33 | 30.40346 | -08.36158 | 1207 | 4 | 2 |
| ARG 34 | 30.40214 | -08.36119 | 1207 | 4 | 2 |
| ARG 35 | 30.40187 | -08.36119 | 1207 | 4 | 2 |
| ARG 36 | 30.40180 | -08.36124 | 1207 | 4 | 2 |

^1^ average altitude of the sampling station.

^2^ with reference to Figure 2.

^3^ used to test measurements errors and repeatability

**Supplementary Table S2.** Presentation of charred wood fragments included in the study.

| Archaeological context | Stratigraphic Unit | Number of charcoal fragments | Accession number |
| --- | --- | --- | --- |
| Gate 1, Entry 1 | 12137 | 5 | 12137-1  12137-2  12137-3  12137-4  12137-5 |
| Plank | 41005 | 1 | 41005 |
| Latrine | 52113 | 5 | 52113-1  52113-2  52113-3  52113-4  52113-5 |
| Beam | 41405 | 4 | 41405-4  41405-7  41405-11  41405-13 |
| Fireplace | 56613 | 5 | 56613-1  56613-2  56613-3  56613-4  56613-5 |

**Supplementary Table S3.** Summary of eco-anatomical data from analysis of modern Argan charred wood samples

| Samples | Sample diameter (mm)^1^ | Surface Vessel area (SVS. µm²) | Density of vessels (DVS, Number/mm²) | Ray density (DRA, Number/mm²) | Axial Parenchyma density (DPA, Number/mm²) | Density of wood fenestrated zones (DWF, Number/mm²) | Surface  of conduction  (SC)^2^ | Vessel conductivity  (CD)^3^ |
| --- | --- | --- | --- | --- | --- | --- | --- | --- |
| *Modern samples* | |  |  |  |  |  |  |  |
| ARG1-1 | 12.71 | 634.22 | 316.77 | 210.32 | 113.11 | 254.02 | 0.20 | 128.66 |
| ARG1-2 | 6.99 | 582.54 | 256.60 | 213.01 | 163.74 | 318.83 | 0.15 | 134.00 |
| ARG2-1 | 12.11 | 857.13 | 198.87 | 218.98 | 109.62 | 192.91 | 0.17 | 374.31 |
| ARG2-2 | 8.50 | 538.60 | 266.34 | 221.50 | 135.32 | 271.07 | 0.14 | 110.35 |
| ARG3-1 | 9.86 | 672.06 | 252.08 | 214.85 | 96.53 | 154.38 | 0.16 | 181.55 |
| ARG3-2 | 17.93 | 704.22 | 212.63 | 232.46 | 138.61 | 232.48 | 0.15 | 236.31 |
| ARG4-1 | 30.61 | 1225.70 | 148.34 | 186.34 | 93.15 | 99.13 | 0.18 | 1026.13 |
| ARG4-2 | 11.75 | 1004.69 | 156.43 | 247.19 | 109.38 | 198.11 | 0.15 | 653.80 |
| ARG5-1 | 12.76 | 654.37 | 194.42 | 210.97 | 93.02 | 151.13 | 0.12 | 223.16 |
| ARG5-2 | 4.40 | 377.64 | 402.85 | 229.48 | 148.28 | 391.12 | 0.15 | 35.87 |
| ARG6-1 | 31.48 | 613.08 | 271.54 | 203.85 | 144.60 | 271.77 | 0.02 | 140.25 |
| ARG6-2 | 15.58 | 711.94 | 195.71 | 246.84 | 131.28 | 236.58 | 0.14 | 262.40 |
| ARG7-1 | 31.93 | 825.10 | 128.36 | 192.44 | 149.97 | 203.44 | 0.11 | 537.37 |
| ARG7-2 | 3.41 | 463.97 | 326.95 | 210.38 | 180.96 | 405.94 | 0.15 | 66.71 |
| ARG8-1 | 31.47 | 521.17 | 114.26 | 191.26 | 126.36 | 198.90 | 0.06 | 240.85 |
| ARG8-2 | 12.33 | 403.98 | 247.51 | 194.83 | 137.72 | 314.96 | 0.10 | 66.81 |
| ARG9 | 8.97 | 666.88 | 318.50 | 212.08 | 128.60 | 212.11 | 0.21 | 141.48 |
| ARG10 | 3.06 | 342.12 | 419.87 | 283.59 | 226.23 | 380.96 | 0.15 | 28.25 |
| ARG11 | 27.80 | 1054.40 | 220.29 | 217.79 | 101.73 | 186.10 | 0.22 | 511.34 |
| ARG12 | 11.83 | 1148.42 | 238.44 | 234.93 | 112.27 | 219.86 | 0.27 | 560.44 |
| ARG13 | 10.11 | 762.93 | 316.23 | 260.43 | 123.48 | 342.92 | 0.24 | 186.50 |
| ARG14 | 14.36 | 911.51 | 233.89 | 157.99 | 114.61 | 201.01 | 0.21 | 359.92 |
| ARG15 | 9.85 | 522.38 | 226.49 | 218.28 | 128.34 | 265.26 | 0.12 | 122.07 |
| ARG16 | 5.42 | 654.84 | 267.37 | 277.01 | 107.09 | 272.47 | 0.17 | 162.50 |
| ARG17 | 14.37 | 593.42 | 408.31 | 269.22 | 121.97 | 327.00 | 0.24 | 87.39 |
| ARG18 | 6.02 | 721.70 | 222.30 | 283.90 | 100.87 | 254.17 | 0.16 | 237.39 |
| ARG19 | 26.49 | 686.11 | 232.38 | 211.43 | 130.50 | 251.79 | 0.16 | 205.25 |
| ARG20 | 53.03 | 891.68 | 186.49 | 194.43 | 135.38 | 234.83 | 0.16 | 431.99 |
| ARG21 | 24.80 | 662.62 | 149.89 | 150.74 | 95.71 | 122.48 | 0.10 | 296.78 |
| ARG22 | 30.89 | 1078.25 | 196.11 | 222.70 | 102.68 | 172.02 | 0.21 | 600.69 |
| ARG23 | 26.62 | 806.83 | 136.35 | 244.83 | 81.47 | 139.54 | 0.11 | 483.73 |
| ARG23 | 11.25 | 782.97 | 212.76 | 219.08 | 72.52 | 173.12 | 0.16 | 291.95 |
| ARG24-1 | 26.40 | 922.18 | 150.99 | 229.83 | 93.78 | 167.94 | 0.14 | 570.67 |
| ARG24-2 | 11.42 | 835.98 | 272.15 | 262.50 | 114.21 | 262.23 | 0.23 | 260.19 |
| ARG25-1 | 25.19 | 958.01 | 151.44 | 207.44 | 93.21 | 182.49 | 0.15 | 614.03 |
| ARG25-2 | 9.34 | 672.37 | 252.36 | 222.28 | 119.92 | 233.45 | 0.17 | 181.50 |
| ARG26-1 | 19.17 | 871.47 | 178.44 | 232.13 | 82.77 | 177.58 | 0.15 | 431.25 |
| ARG26-2 | 10.37 | 738.34 | 253.47 | 242.33 | 129.49 | 249.23 | 0.19 | 217.91 |
| ARG27 | 17.00 | 1054.21 | 230.15 | 209.53 | 144.09 | 290.60 | 0.23 | 489.26 |
| ARG28 | 4.90 | 471.11 | 367.15 | 248.25 | 184.62 | 440.18 | 0.17 | 61.25 |
| ARG29 | 7.17 | 680.34 | 369.51 | 246.72 | 88.32 | 192.50 | 0.25 | 126.92 |
| ARG30 | 4.21 | 348.70 | 362.66 | 248.61 | 194.77 | 417.51 | 0.12 | 33.97 |
| ARG31 | 15.25 | 805.58 | 273.25 | 243.53 | 113.51 | 272.59 | 0.22 | 240.63 |
| ARG32-1 | 11.97 | 1040.67 | 150.65 | 208.85 | 160.78 | 172.67 | 0.16 | 728.38 |
| ARG32-2 | 7.84 | 784.05 | 235.71 | 210.93 | 149.90 | 315.21 | 0.18 | 264.24 |
| ARG33-1 | 20.97 | 993.54 | 144.92 | 222.99 | 94.57 | 145.44 | 0.14 | 690.15 |
| ARG33-2 | 8.80 | 893.30 | 203.89 | 237.72 | 113.39 | 224.85 | 0.18 | 396.55 |
| ARG34-1 | 17.82 | 739.81 | 93.63 | 174.64 | 108.03 | 137.89 | 0.07 | 592.28 |
| ARG34-2 | 8.67 | 844.18 | 169.27 | 207.21 | 101.20 | 156.34 | 0.14 | 426.57 |
| ARG35-1 | 25.86 | 789.85 | 106.65 | 226.12 | 73.16 | 140.52 | 0.08 | 592.70 |
| ARG35-2 | 10.91 | 875.78 | 206.53 | 205.32 | 112.85 | 222.34 | 0.18 | 376.27 |
| ARG36-1 | 21.87 | 926.04 | 140.13 | 175.87 | 81.62 | 128.82 | 0.13 | 620.06 |
| ARG36-2 | 7.31 | 961.42 | 199.40 | 204.89 | 111.66 | 191.82 | 0.19 | 469.68 |
| *Test samples* |  |  |  |  |  |  |  |  |
| ARG8B-2-OP1 | 12.33 | 406.54 | 242.67 | 199.54 | 140.23 | 333.33 | 0.09 | 69.01 |
| ARG8B-3-OP1 | 12.33 | 405.4 | 245.64 | 196.09 | 133.87 | 322.09 | 0.1 | 67.79 |
| ARG8B-4-OP2 | 12.33 | 409.22 | 233.85 | 190.01 | 137.64 | 315.44 | 0.11 | 72.56 |
| ARG8B-5-OP2 | 12.33 | 402.37 | 241.78 | 192.97 | 141.08 | 309.87 | 0.1 | 67.85 |
| ARG8B-2-OP1 | 12.33 | 406.54 | 242.67 | 199.54 | 140.23 | 333.33 | 0.09 | 69.01 |
| *Archaeological samples* | | | | | | | | |
| 12137-1 | - | 1335.80 | 219.05 | 192.02 | 99.57 | 132.90 | 0.29 | 825.36 |
| 12137-2 | - | 1058.08 | 203.66 | 172.78 | 126.00 | 154.86 | 0.21 | 556.97 |
| 12137-3 | - | 1066.52 | 194.40 | 216.64 | 59.47 | 119.91 | 0.21 | 592.85 |
| 12137-4 | - | 1292.05 | 239.56 | 190.03 | 97.00 | 126.08 | 0.31 | 706.06 |
| 12137-5 | - | 1158.37 | 206.37 | 203.62 | 91.66 | 174.35 | 0.24 | 658.78 |
| 41005 | - | 1550.17 | 101.85 | 196.73 | 94.93 | 127.68 | 0.16 | 2390.61 |
| 52113-1 | - | 1579.80 | 158.38 | 164.56 | 87.61 | 138.30 | 0.25 | 1596.58 |
| 52113-2 | - | 1545.68 | 169.43 | 171.21 | 117.28 | 131.11 | 0.26 | 1428.70 |
| 52113-3 | - | 912.99 | 183.38 | 200.32 | 73.37 | 150.50 | 0.17 | 460.55 |
| 52113-4 | - | 1220.37 | 187.43 | 170.10 | 91.86 | 104.94 | 0.23 | 805.07 |
| 52113-5 | - | 1352.18 | 192.07 | 218.91 | 118.98 | 151.72 | 0.26 | 964.52 |
| 41405-4 | - | 1586.57 | 120.54 | 227.79 | 126.18 | 166.17 | 0.19 | 2115.84 |
| 41405-7 | - | 1080.15 | 116.01 | 254.39 | 75.14 | 112.97 | 0.12 | 1019.01 |
| 41405-11 | - | 1374.66 | 157.57 | 233.48 | 97.95 | 128.70 | 0.22 | 1215.10 |
| 41405-13 | - | 1196.99 | 118.18 | 194.30 | 89.26 | 115.67 | 0.14 | 1228.38 |
| 54613-1 | - | 1020.46 | 170.96 | 184.94 | 94.47 | 115.43 | 0.17 | 617.14 |
| 54613-2 | - | 1270.29 | 214.30 | 230.91 | 102.76 | 142.16 | 0.27 | 762.93 |
| 54613-3 | - | 788.95 | 111.61 | 305.12 | 108.55 | 190.06 | 0.09 | 565.05 |
| 54613-4 | - | 1192.45 | 185.19 | 248.99 | 71.52 | 124.37 | 0.22 | 777.98 |
| 54613-5 | - | 970.66 | 197.95 | 268.85 | 145.52 | 222.67 | 0.19 | 482.25 |

^1^ Measured before charring; ^2^ SC=Conductive surface/total wood area; ^3^ CD=(SVS/π)^2^/DVS (Terral and Mengüal, 1999; Terral et al., 2004)

**Supplementary Figure S1.** Cumulative average curves allowing us to estimate for ARG1-1, the number of measurements required (dashed segment) for a reliable estimate of an anatomical character. The number of measurements required for an optimal assessment of anatomical features is: 50 measurements for Vessel surface area (SVS), 10 for Vessel density (DVS), 15 for ray density (RDA), 15 for Axial parenchyma density (DPA) and 10 for density of wood fenestrated zones (DWF), as shown in rarefaction curves or cumulative average curves (Fig. S1).


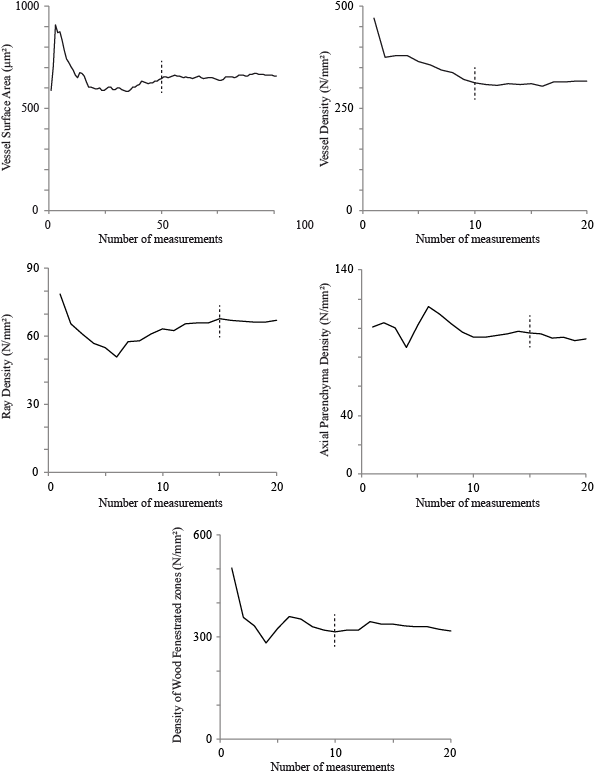


**Additional information about “Measurement errors and reproducibility of measurements”**

As shown in the “Variation of wood anatomical features according to environmental parameters” section, location in the PCA1-3 of the 4 repeated measurements carried out by the two different operators on the ARG8-2 sample, do not show significant differences in comparison of the initial quantitative anatomical data (Fig. 6). As a result, measurement errors are minimal and the eco-anatomical procedure is repeatable.
